# Management and Outcomes of Patients at a Specialty Clinic for *Clostridioides difficile* Infection

**DOI:** 10.1093/ofid/ofae153

**Published:** 2024-03-15

**Authors:** Aaron Hunt, Larry H Danziger, Stuart Johnson, Andrew M Skinner

**Affiliations:** University of Illinois at Chicago, College of Pharmacy, Chicago, Illinois, USA; University of Illinois at Chicago, College of Pharmacy, Chicago, Illinois, USA; Edward Hines Jr., VA Hospital Research Service, Hines, Illinois, USA; Loyola University, Stritch School of Medicine, Maywood, Illinois, USA; George E Wahlen VA Hospital, Research Service, Salt Lake City, Utah, USA; University of Utah, School of Medicine, Salt Lake City, Utah, USA

**Keywords:** bezlotoxumab, *Clostridioides difficile*, fidaxomicin, recurrent *C. difficile* infection, vancomycin

## Abstract

Vancomycin and fidaxomicin taper regimens were the most common treatment strategies employed but nearly half of patients (40/83) referred to our *Clostridioides difficile* infection (CDI) clinic did not require further treatment. The overall 60-day CDI recurrence rate was 16.9% (11/65). CDI management at a dedicated clinic may improve clinical outcomes.

## BACKGROUND

Recurrent *Clostridioides difficile* infections (rCDI) are difficult to treat and have necessitated the development of numerous treatment strategies [1–4]. Patients with multiple recurrences have an estimated 40%–65% risk of future recurrence [5]. Recently published guidelines from the Infectious Diseases Society of America and the Society for Healthcare Epidemiology of America favor the use of fidaxomicin because of lower rates of recurrence but noninferior rates of initial clinical cure when compared with vancomycin [2]. Taper and pulse regimens of vancomycin and fidaxomicin, which alter the dose and duration of antibiotics compared with labeled directions for initial infection, are a common empiric strategy for managing patients with rCDI [6–8]. Guideline recommendations to treat rCDI also include the use of bezlotoxumab and fecal microbiota transplant [2–4]. During their clinical course, patients with rCDI commonly require multiple or repeated therapies to achieve sustained clinical cure [5]. Thus, expert management could aid in addressing issues of recurrence and improve clinical outcomes [9, 10].

## METHODS

We performed a retrospective case series of new patient encounters presenting to the Loyola University *C difficile* clinic from 1 January 2021 to 31 December 2022. Patients were included for analysis if they had never been seen in the clinic or if they had not been seen in the clinic for ≥1 year. The biweekly clinic was staffed by 2 infectious diseases physicians with a combined 40 years’ experience in the management of CDI and by an infectious diseases pharmacist.

Each chart was reviewed for age, sex, ethnicity, gastrointestinal comorbidities, and immunocompromising conditions. Episodes were characterized by primary versus recurrent infection, symptoms at time of appointment, and prior therapies used. Recurrence was defined as a symptomatic episode with a positive *C difficile* assay within 8 weeks of a previous episode of CDI. The primary *C difficile* assay used at Loyola was polymerase chain reaction (GeneXpert, Cepheid, Sunnyvale, California) but assays ordered by an outside institution were considered for past recurrences. Past recurrences, prior treatment regimens, and laboratory tests for *C difficile* available before the initial appointment were reviewed. This information was used in conjunction with documented rationale to categorize patient therapy selection.

The primary outcome was to describe treatment selection at initial patient presentation to the specialty clinic. Secondary outcomes included the 60-day recurrence rates by number of prior CDI episodes and treatment decisions. A total of 18 patients were excluded from 60-day recurrence analysis because they were either lost to follow-up (n = 17) or placed on long-term vancomycin suppression (n = 1). Descriptive statistics were provided for all categories and χ^2^ test was completed when comparing key variables.

This study was reviewed and deemed exempt by the institutional review board (LU215632).

## RESULTS

From 1 January 2021 to 31 December 2022, we identified 83 unique patients that presented to the *C difficile* clinic. The median age of participants was 65 years (interquartile range 58.5–73) and 59% (49/83) were female. Gastrointestinal comorbidities were documented in 18% (15/83) of patients and 25% (21/83) were immunocompromised. The majority of patients (75%, 62/83) presented to the clinic with recurrent infection (≥1 CDI episode) and 40% (33/83) of patients had 2 or more recurrences by the time they were seen in clinic. Patients were asymptomatic at the time of initial clinic presentation in 26.5% of cases (22/83). Approximately 50% of patients (44/83) were on some form of treatment for CDI at the time of their appointment. Based on clinical evaluation, 39% (32/83) were considered to have a questionable CDI diagnosis once the history of present illness was evaluated.

**Figure 1.**
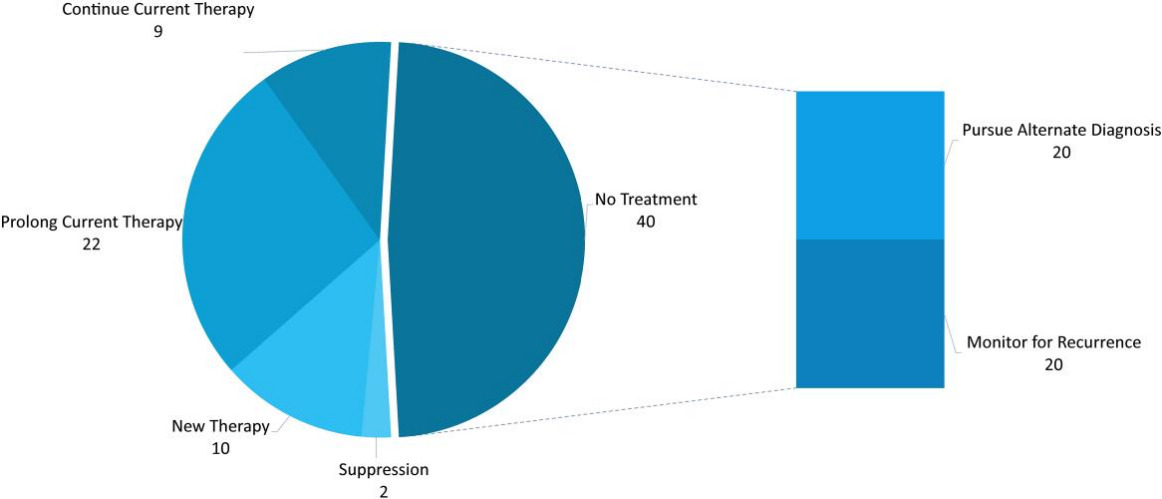
Treatment decisions at patient presentation. Continue current therapy: Patients continue medication therapy started by outside provider without changes. Prolong current therapy: Duration of patient medication therapy extended. Monitor for recurrence: Patients did not receive treatment and did not have a questionable diagnosis. New therapy: Drug therapy changed or new regimen initiated. No treatment: Drug therapy discontinued or not started, depending on patient presentation. Pursue alternate diagnosis: Patients did not receive treatment and had a questionable diagnosis documented. Suppression: Vancomycin suppression therapy started until resolution of temporary risk factors.

Nearly half of patients (40/83) were deemed to not require treatment and either had therapy discontinued or were monitored for recurrence, as appropriate. Among these 40, 50% were recommended to pursue an alternative diagnosis for their diarrheal illness. The other 50% were recommended to monitor for CDI recurrence after having completed therapy. Nine of 83 patients demonstrated an appropriate response to currently administered therapy and were continued on the regimen prescribed by outside providers. The most common decision when modifying patient treatment was to prolong current therapy by prolonging the taper of the antibiotic (vancomycin or fidaxomicin) the patient was receiving at the time of evaluation (Figure 1).

Among individuals who were no longer on CDI treatment and who were asymptomatic, all were monitored off CDI therapy (22/83). Among those that were off CDI therapy and presented to the clinic with diarrhea, 64.7% (11/17) were recommended to pursue an alternative diagnosis, and 29.4% (5/17) were started on new CDI therapy. One person was placed on long-term suppression because of a history of untreated ulcerative colitis that symptomatically improved with vancomycin.

Among the 44 persons who presented to the clinic while still on treatment, 50% (22/44) had their treatment extended to a prolonged taper, 20.5% (9/44) were continued on their current regimen, 11.3% (5/44) had their therapy changed to another antibiotic, and 15.9% (7/44) were taken off of CDI therapy and monitored. Additionally, 1 patient was placed on long-term suppression to provide prophylaxis during planned urinary and dental procedures when systemic antibiotics would be administered. This patient's history of multiply recurrent CDI in 2019 following antibiotic exposure for recurrent urinary tract infections contributed to this decision.

Among the 43 persons in which continuation of CDI therapy occurred, the majority (32/43) were given taper-based regimens. Vancomycin and fidaxomicin tapers, as previously described, accounted for 56% and 44% of the taper regimens, respectively [6, 7]. Adjunctive bezlotoxumab was used in 16.3% (7/43) of patients as previously described [11]. A standard vancomycin course was only used in 2 patients.

After excluding 17 patients who were lost to follow-up and 1 patient who was placed on long-term CDI suppression, the overall CDI recurrence rate within 60 days was 16.9% (11/65) (Table 1). Among the individuals who presented to the clinic and the decision to not treat further for CDI was recommended, 15.2% (5/33) developed an rCDI. In comparison, among patients that were treated for CDI, 18.8% (6/32) developed recurrence within 60 days (*P* = .699). Similar recurrence rates were seen for each therapy selected, with 23.1% (3/13) for vancomycin taper, 18.2% (2/11) for fidaxomicin taper, and 16.7% (1/6) with adjunctive bezlotoxumab. Patients who presented to the clinic with a primary CDI episode trended toward a higher proportion of recurrence: 22.2% developed a recurrent episode within 60 days and 14.6% of individuals with recurrent CDI had from a subsequent recurrence (*P* = .46).

**Table 1.** 60-Day Clinical Outcomes Among Evaluable Patients^a^

	Recurrence	Resolution
Overall	11/65 (16.9%)	54/65 (83.1%)
Treatment decision		
No treatment	5/33 (15.2%)	28/33 (84.8%)
* Pursue alternative diagnosis*	2/13 (15.4%)	11/13 (84.6%)
* Monitor for symptoms*	3/20 (15%)	17/20 (85%)
Treatment provided	6/32 (18.8%)	26/32 (81.3%)
Standard vancomycin^b^	-	2/2 (100%)
Vancomycin taper	3/13 (23.1%)	10/13 (76.9%)
Fidaxomicin taper	2/11 (16.7%)	9/11 (83.3%)
** **Adjunctive bezlotoxumab	1/6 (16.7%)	5/6 (83.3%)
By episode		
Initial	4/18 (22.2%)	13/17 (76.4%)
First recurrence	4/24 (16.7%)	20/24 (83.3%)
Second recurrence	2/14 (14.3%)	12/14 (85.7%)
Third or greater recurrence	1/10 (10%)	9/10 (90%)

^a^Excluded 17 patients from loss of follow-up and 1 patient on vancomycin suppression.

^b^Vancomycin 125 mg 4 × daily × 10 d.

## DISCUSSION

The management of patients with CDI first begins with a careful assessment of the presumed diagnosis. In our clinic, each patient was evaluated for symptom onset, stool frequency, stool description, baseline bowel habits, and response to prior treatments. Nearly half (40/83) of patients referred did not require further treatment for CDI at the time of presentation to the specialty clinic. Symptoms elicited from many of these patients suggested an etiology other than CDI: intermittent loose stools, diarrhea limited to certain times of the day, and symptoms unresponsive to standard-of-care antibiotic therapy for CDI. Careful evaluation of patients referred to the clinic identified patients requiring workup for alternative etiologies and limited unnecessary additional antibiotic exposure with CDI antibiotics.

Vancomycin was the most common treatment selection because of either concerns of affordability or patients already receiving vancomycin at the time of appointment. Although recommended by the 2021 Infectious Diseases Society of America guidelines, high copay cost, or lack of insurance coverage limits fidaxomicin use in clinical practice [12]. Several patients in this study required patient assistance program enrollment to gain access to fidaxomicin. Adjunctive bezlotoxumab was recommended for 7 patients because of either severity of prior CDI episodes or failure of multiple previous taper regimens. The administration of this agent required coordination with infusion centers and insurance to determine scheduling and coverage. Pharmacy specialists aid in the selection of ideal regimens to limit recurrence and may reduce delays in medication access because of familiarity with product coverage and available patient resources [13].

The majority of patients seen in the specialty clinic presented with recurrent infection. Despite this fact, rates of recurrence were much lower than literature estimates of 40% or greater [5]. Patients selected to not receive further treatment had similar rates of recurrence to other treatment decisions. No difference in recurrence was seen between those who had a suspected alternate diagnosis and those who only required monitoring. This finding supports our determination of which patients likely did not have had true CDI. In addition, stopping treatment potentially spared further fecal microbiota disruption associated with CDI antimicrobial therapies [14]. In summary, these findings support the importance of a specialty CDI clinic dedicated to outpatient follow-up to limit subsequent recurrences even among patients with multiple prior CDI recurrences.

This study has several limitations, including the limited sample size of the population. Seventeen patients were lost to follow-up or referred to care with inaccessible records, further limiting outcome determinations. Additionally, the nature of this study necessitates simplifying patient information into categories and limits the ability to communicate nuances that informed clinical decision making. Fecal microbiota transplant was not part of therapeutic decisions in our clinic for reasons of product limitation during the COVID-19 pandemic, lack of a Food and Drug Administration–approved product, and our management preferences. Despite these limitations, this study offers valuable insight into the decision making of CDI specialists and critical CDI outcomes. Expert management of CDI through a dedicated specialty clinic has the potential to improve patient outcomes for recurrent *C difficile* infections.

## Notes

***Author Contributions.*** All authors contributed to project design, data collection, data analyses, and manuscript preparation.

***Patient consent statement.*** Patient consent was deemed not to be required as an exempt study.

***Data availability.*** Data not publicly available.

***Financial support.*** A.M.S. and S.J. receive funding through the United States (U.S.) Veteran Affairs Research and Development. This project was supported in part by Career Development Award #IK2BX005609 from the U.S. Department of Veteran Affairs Biomedical Laboratory Research and Development Service. Content is solely the responsibility of the authors and does not necessarily represent the official views of the United States Veteran Affairs or US Government.
